# Patients’ experience of being triaged directly to a psychologist in primary care: a qualitative study

**DOI:** 10.1017/S1463423613000339

**Published:** 2013-08-30

**Authors:** Linda Dahlöf, Anna Simonsson, Jörgen Thorn, Maria EH Larsson

**Affiliations:** 1 Clinical Psychologist, Primary Health Care, Region Västra Götaland, Sweden; 2 Department of Public Health and Community Medicine, Sahlgrenska Academy, University of Gothenburg, Primary Health Care, Region Västra Götaland, Sweden; 3 Department of Clinical Neuroscience and Rehabilitation/Physiotherapy, Institute of Neuroscience and Physiology, Sahlgrenska Academy, University of Gothenburg, Primary Health Care, Region Västra Götaland, Sweden

**Keywords:** assessment, patient experience, primary health care, psychologist, triage

## Abstract

**Background:**

In a primary health-care centre (PHCC) situated in a segregated area with low socio-economic status, ‘primary care triage’ has increased efficiency and accessibility. In the primary-care triage, the nurse sorts the patient to the appropriate PHCC profession according to described symptoms.

**Aim:**

The aim of this study was to examine the patients’ experience of being triaged directly to a psychologist for assessment.

**Method:**

Interviews were conducted with 20 patients and then analysed using qualitative content analysis.

**Findings:**

The results show that patients contacting the PHCC for mental health issues often are active agents with their own intent to see a psychologist, not a doctor, as a first-hand choice when contacting the PHCC. Seeking help for mental health issues is described as a sensitive issue that demands building up strength before contacting. The quick access to the preferred health-care professional is appreciated. The nurse was perceived as a caring facilitator rather than a decision maker. It is the patient's wish rather than the symptoms that directs the sorting. The patients’ expectations when meeting the psychologist were wide and diverse. The structured assessment sometimes collided and sometimes united with these expectations, yielding different outcome satisfaction. The results could be seen in line with the present goal to increase patients’ choice in the health-care system. The improved accessibility to the psychologist seems to meet community expectations. The results also indicate a need for providing more prior information about the assessment and potential outcomes.

## Introduction

Patients who seek health care for mental health issues have to wait longer for diagnosis and treatment than patients seeking regular health care; meanwhile, they seek more regular health care than the general population (Walker and Collins, 2009). The majority of patients seeking help for mental health issues do so at a primary-care level (Kessler, 2009). In 2008, 30% of the visits to Swedish primary care were by patients with mental health issues (Nordstrom and Bodlund, 2008) and this level keeps rising (Socialstyrelsen, 2010). In Sweden and other European countries, primary care has been given an increased responsibility to manage this patient group, with the incentive to improve access and early intervention (van Orden *et al.*, 2009; Harkness *et al.*, 2010; Socialstyrelsen, 2010; NICE, 2011).

The National Board of Health and Welfare in Sweden recommends evidence-based psychotherapy and medication for treating mild to moderate depression and anxiety disorders, with psychotherapy being the first-hand choice for certain diagnoses (Socialstyrelsen, 2010). However, in the present Swedish primary care, medication is the most common treatment for mental disorders and only every third patient receives psychological treatment, most often through a general practitioner's (GP) referral (Socialstyrelsen, 2007). Studies show that GPs oversubscribe antidepressants (Smith *et al.*, 2003; Åsbring and Hochwälder, 2009), fail to refer to psychologists (Walker and Collins, 2009), and tend to refer patients with disorders for which psychotherapy has low evidence (Henninger, 2009). Meanwhile, patients request psychotherapy in primary care (Seligman, 1995; Dwight-Johnson *et al.*, 2001; Socialstyrelsen, 2007; Walters *et al.*, 2008), but would at the same time primarily contact medical health professionals when seeking mental health care (Gunn and Blount, 2009). With this background, it is important to examine the possibility of psychologists making the first-line assessment and evaluating the patient's suitability for psychological treatment.

The primary health-care centre (PHCC) where this study was conducted is situated in a segregated suburb with low socio-economic status in Gothenburg, Sweden. With the intention of increasing the accessibility to health care at the correct professional level, a structured patient-sorting system called ‘Primary Care Triage’ was introduced at the PHCC in 2008. Following an instruction manual, patients are sorted by a nurse to the appropriate professional category (GP, physiotherapist, district nurse, psychologist) on the basis of symptoms (Thorn *et al.*, 2010).

Primary-care triage has led to enhanced efficiency and increased accessibility to all occupational groups, including psychologists (Thorn *et al.*, 2010), but it has not yet been evaluated from a user's perspective. The most common request by patients is to see a GP (Häger Glenngård and Anell, 2012), and therefore it was interesting to see how patients experience meeting another health-care professional. The purpose of this study was to describe the patient's experience of being triaged directly to a psychologist for assessment when the reason for contact is mental health issues.

## Method

### Sample

The informants in this study were recruited from the patients who had been triaged directly to a psychologist's assessment between July 2010 and July 2011 through the primary-care triage (Thorn *et al.*, 2010) at the PHCC mentioned above (*n* = 142). Of these, 94 patients met the inclusion criteria; a nurse had booked the patient to a psychologist's assessment and the patient had not been booked to another professional for the same symptoms. Following the recommendation by Kvale (1996) to interview enough subjects to enable generalizations, yet manage to thoroughly interpret the data, it was decided to include 20 patients in the study. The 45 patients that had been triaged most recently were contacted first, following the assumption that the experience could be described in greater detail the more recently it had occurred. Of these, three patients were excluded: two because of severe mental ill health and one patient had deceased. In addition to selection by date, strategic selection was made to enhance representativeness. For example, extra effort was made to include male patients and patients with foreign background. The 20 interviews, with 14 women and six men, were completed among the first group of 45 patients, and therefore others were not contacted. The informants’ age varied from 21 to 53 years. The average age was 30 years. Five informants had foreign background, that is, the informants parents were born in a foreign country (Statistics Sweden, 2007). Of the 20 informants, five had a depression diagnosis and 11 had an anxiety diagnosis, two informants had both depression and anxiety diagnosis, and two of the informants were not diagnosed.

### Data collection

Patients were sent letters with information about the study. They were then contacted by telephone within two weeks following the letter, to enquire about their willingness to participate. They were ensured that their participation was voluntary and that possible future treatment would not be affected. Semi-structured interviews were made individually at a location nearby the PHCC, each taking 20–45 min to conduct. The informants were interviewed by a pre-graduate psychologist with previous experience of interviewing for qualitative research. One pilot interview was conducted in September 2011. The remaining interviews were conducted in September–October 2011. The pilot interview was included in the study. The main topics addressed during the interview followed the chronology of the triage: to take the step to contact the PHCC for mental health issues, to be triaged by a nurse, and to be assessed by a psychologist. The purpose of having topics was to guide but not dictate the interview (Willig, 2001). The interviews were tape-recorded and transcribed verbatim. It was discovered that one of the informants did not meet the correct inclusion criteria, as the patient had been booked to a doctor for the same symptoms. The interview was still included in the study as the patient had been immediately redirected to a psychologist.

### Data analysis

To search for the central yet subjective content of the patients’ experience in a non-prejudicial manner, the study has a phenomenological approach (Zahavi, 2003; Krippendorff, 2004). The qualitative method, with open-ended interviews, was considered most appropriate to be able to gather data without imposing too much structure on subjects (Krippendorff, 2004). The data, the text transcribed from the interviews, was analysed using thematic qualitative content analysis, according to Graneheim and Lundman's (2004) model. The thematic analysis involved the identification of codes, categories, and themes. The computer software package, NVivo 9 (QSR International Pty Ltd, Doncaster, Victoria, Australia), was used to organize the data. There were no predefined definitions. Instead, categories and themes were found in an inductive approach. Each interview was read and systematically analysed for meaning units by the first two authors. At first, the analysis was performed together to get a mutual understanding and concept of the material. Later in the process, the analysis was also performed individually by the first two authors, with continuous discussion and feedback between them. The meaning units found were condensed into codes, staying close to the original descriptive data. The codes were compared with each other and abstracted into 12 different categories. The overarching meanings of the categories were abstracted into three themes. The reliability of the analysis was additionally supported through continuous discussion with the last author, who is more experienced in the method.

The study was approved by the Regional Ethical Review Board in Gothenburg.

## Results

Three themes were found, following the chronology of the triage: to contact the PHCC, to be booked to a psychologist by a nurse, and to be assessed by the psychologist (see Table 1). The results suggest that patients are active agents who themselves have considered different types of treatment and have their own intent to see a psychologist when contacting the PHCC. Seeking help for mental health issues is done during a critical time period where patients need to build up strength before contact. Therefore, they much appreciate the easy access to see a psychologist. The structured assessment sometimes collided and sometimes united with the patients’ expectations. Below, the three themes are described in more detail.

**Table 1:** Themes and categories

Triage	Category	Theme
Contacting	To seek help for mental health issues is demanding	Appreciating quick access as seeking help is
	Disturbing preconceptions about psychologists	demanding
	Appreciating easy access because of critical needs	
Booking	Patients are informed active agents	The nurse responds to the patient's own intention
	Nurse as a caring, non-judgemental assistant	
Assessment	Appreciating attentive care and space	Relief and disappointment when diverse
	Relief and effect from assessment	expectations meet the structured assessment
	Frustration over information and communication difficulties	
	Disappointing discrepancy between experienced need and offered care	
	Relying on the psychologist as a professional	
	Collaborative approach	
	Rejecting assessment	

### Appreciating quick access as seeking help is demanding

This theme describes the experiences of contacting the PHCC for mental health issues. The categories included are: *To seek help for mental health issues is demanding, Disturbing preconceptions about psychologists*, and *Appreciating easy access because of critical needs*.

*To seek help for mental health issues is demanding*. Patients describe a feeling of heightened vulnerability when seeking help, which means having to admit to oneself that there is a problem. It is a difficult and sensitive matter to talk about mental health issues and the informants were often nervous before contacting. While procrastinating to seek help, the problems can increase to a crucial limit. Seeking help seems to be done in a burst of effort during a critical period. There are both very high hopes and very negative expectations, sometimes present within the same informant. To have booked an appointment often gives a positive effect in itself, as it eases the worry about being rejected.

Int 8: …I constantly had precisely those feelings – she might think that I'm silly to have come there at all.

The negative anticipations are connected to uncertainties around what help there is within the health-care system. There is an expectation of having to ‘fight the system’ to actually get noticed and receive help.

Int 20: … and I've sort of had to fight my way, to really prove that I need help, and with new contacts in health care it feels like… Here one has to be loaded like a gun to really get noticed, and slam my fist on to the table… I need help! I'm not going to leave until I get the help I need!

The informants expected obstacles, for example, waiting lists, which are considered extra straining when feeling mentally ill. They also worried that it would be more difficult to get help for psychological problems than somatic.

Part of the hesitation when seeking help concerns *disturbing preconceptions about psychologists*, their role, and agenda. These are somewhat caricatured images influenced by popular culture such as films and TV shows, which can aggravate the help-seeking process. There are expectations that the psychologist's role is to be quiet, to question what the patient says, that the psychologist can perceive everything, and/or that no advice would be given.

Int 3: They sit quietly and ask counter questions all the time. It wasn't like that of course, but that is what one thinks. […] One thinks that it will be someone that doesn't really understand, who just sits there in silence, taking notes on everything one says. But it wasn't really like that, fortunately, like that image one has from movies.

*Appreciating easy access because of critical needs*. The informants describe a positive experience of easy access and a low threshold to get an appointment with the PHCC psychologist. They are surprised and appreciative that there are no detours. There is a positive feeling of having been given own agency:

Int 1: For me that felt good. It would feel unnecessary to have to talk to somebody else first to see if one would be suitable to go to a psychologist…

The flow in the process is considered especially important due to the momentum described above when taking the step to seek help. It is stressed that it is important that needs are met readily on all levels of health care during this open window of opportunity, for it to be helpful and followed through. If successful, the burst of effort can give a proud and positive feeling of having taken charge of the problem.

### The nurse responds to the patient's own intention

This theme covers the experiences of being booked to a psychologist by the nurse. The categories included are: *Patients are informed active agents* and *Nurse as a caring, non-judgemental assistant*. Informants describe that they had their own intention to see a psychologist and they experienced the nurse as being a caring professional, assisting them in their request.

*Patients are informed active agents* who have prior knowledge about available mental health care, including the possibility to seek psychological treatment. This knowledge is attained through either of their own prior experience, friends, family, internet, and/or phone inquiries. The informants describe how they actively prepare what to say to the nurse to access a psychologist's appointment, not having to describe too much or too little. The alternative to see a doctor first is considered a detour. They are assertive in their decision to get a psychologist's appointment and many also want to influence the treatment.

Int 3: Yes she listened to what I wanted, I told her the suggestion and she listened to it. She thought that was the best too. It was probably because I was so targeted and already knew…

The informants perceive the *nurse as a caring, non-judgemental assistant*, a facilitator in the booking procedure. The nurse's approach is described as professional, warm, and secure. This is often described as important in itself, providing energy and strength to continue the help-seeking process. When expressing their needs, the informants find the nurse to give an empathetic, pithily answer – not asking too much or too little.

Int 17: P: Very warm, very lovely…you know, very secure in their professional roles and it was as if they had had lots of education in just making people feel safe and well.

The nurse is perceived as non-judgemental and equal, sometimes in contrast to the psychologist who had a more evaluating role. The nurse is not perceived as making the decision to book a psychologist's appointment. Instead, the informants view the booking as guided by their own wish and preference, assisted by the nurse.

### Relief and disappointment when diverse expectations meet the structured assessment

This describes the experiences of being assessed by the psychologist. The categories included are: *Appreciating attentive care and space,* *Relief and effect from assessment*, *Frustration over information and communication difficulties,* *Disappointing discrepancy between experienced need and offered care, Relying on the psychologist as a professional, Collaborative approach, and Rejecting assessment*. The informants express a broad spectrum of ideas about the role of a psychologist and a patient, as well as the content and outcome of the treatment. These ideas are sometimes colliding, both between and within individuals. As described above, informants come to the psychologist's appointment with high tension, expectations, and fear of rejection. This fuels strong reactions, positive and negative, over the structured assessment.

*Appreciating attentive care and space*: many informants describe a positive feeling of being accepted and taken seriously during the assessment that gives some relief. It is described as vital to have been given enough space and time in a calm environment. The psychologist is experienced as giving full attention, being understanding and a good listener, making it easier to talk about oneself.

Int 2: Well, I felt very sure about getting an appointment this time around, just how it was a fairly urgent time-booking since there was not a lot of in-between time so, well it didn't feel like I was somehow intruding on something else… there was time here and that time was for me […] I was happy to get there so soon and be taken seriously and then to get space…

Informants describe *relief and effect from assessment*, they are pleased, and sometimes surprised, over the instant ease they feel. Others express the notion of having started a changing process. The assessment is described as both focused on solution and learning more about oneself. Receiving advice and/or suggestions about further steps to take is appreciated and connected to the informants’ feeling of improvement after the first assessment.

Int 13: …honestly….she could fix it in an hour. A thing that I have carried around you know for many years…through that she listened you know… She, who I had never met before… she could start to solve it in an hour…she could tell me what it is I really need to do…

Informants who are more disappointed describe *frustration over information and communication difficulties*. There is a difficulty to describe mental issues and deep personal concerns accurately. Extra limitation is experienced by those who do not have Swedish as their native language.

Int 16: …she explained in a way I did not always understand… and I often had to ask – I'm not sure what you mean […] well, it was a bit complicated for me.

Flaws in communication are also attributed to the psychologist, who is found hard to understand or out of tune. They also express a need for a clearer structure and more information beforehand, during the assessment and about alternative outcomes.

Some also experience a *disappointing discrepancy between experienced need and offered care*, resulting in no effect or even feeling worse. They want more, sometimes more than what can be considered regular health care. A feeling that time is too limited is a recurring complaint.

Int 12: …considering she started the conversation with…. You have 50 minutes…It felt like …shit…now I really have to choose what I'm going to say […] I don't know, but I think you have to be careful if you are the one who's going to sit and listen to the one who has problems that eh sometimes it might take half an hour…sometimes it might take two hours…you have to give it some time…because the person that enters, like me for example… I was really feeling *terrible*…

Informants are also disappointed with the psychologist's lack of skill and feedback. The suggestion of transfer to another caregiver can also be disappointing and the feeling of rejection can be strong.

Int 17: but all that was contaminated by the betrayal I felt when she sent me off to a clinic for people with addiction-problems, which I didn't think I had…

There seem to be diverging expectations and experiences of the patient's and the psychologist's role. These are again sometimes verified and sometimes disappointed. Some informants emphasize *relying on the psychologist as a professional* as an important aspect of the assessment. It is stressed that it is even more difficult to make decisions for oneself owing to the mental state. There is a notion that the psychologist can discover new things and that the problems were affirmed, explained, and described as solvable. As patients rely on the psychologist as an expert, they do not feel the same need to influence the outcome. The informants appreciate that the first session was an assessment, taking comfort in the psychologist being active and practised. It is a relief that it is a job, not a personal investment for the psychologist.

Int 10: Well, I guess it felt more safe because of the thought that…at least I suppose that the psychologist has a special competence within her area […] To me it is easier to be objective around a physical complaint […] but a mental is a little more sensitive to me and there is a big difference between going to see a psychologist than a doctor I find.

Some informants describe a *collaborative approach* to the assessment, a mutual exchange of knowledge, where the patient has his/her own responsibility and motivation. The patient has a clear objective throughout the process. The assessment is perceived as a gateway to different outcomes, a means to an end. If the assessment feels successful, it is explained more by the patient's own knowledge and skill to navigate the system. It is more of an experience for the psychologist, following the patient, as opposed to the experience described in the previous category.

Int 9: and that's what it was about and we solved it the best we could and that was that, so it was very efficient.

Some informants seem to be *rejecting assessment*; it was not what they aimed for. It is stressed that the nature of their complaints demanded something other than a clinical assessment. The reasons for rejecting are very different. It can sometimes be because of a wish to get straight to the interventions.

Int 19: and maybe go straight to the problem instead of just analysing what had happened, maybe to more get started with something…with thought exercises or something of the kind.

For others, the main objective of the informant was to get support and comfort from an equal listener. They express that they just wanted to unload, talk about the general life situation and not just a specific problem.

Int 15: Really…if I could just have been accepted….and really if she had said….Just tell me how it feels….how are you…just nodded…maybe just listened really…been a little more soft so to say…a little more gentle…that is what I was missing…

Standardized evaluation forms are criticized for making it more impersonal. It is expressed that the assessment creates a negative hierarchy between the patient and the psychologist.

The informant reacts against the psychologist being active and taking a professional stance, experienced as too clinical for the patient.

## Discussion

To the best of our knowledge, this is the first study to describe patients’ experience of being triaged to a psychologist. Traditionally, a doctor's appointment has been the most important factor for patient's satisfaction in primary care (Häger Glenngård and Anell, 2012). Following this, one major topic the authors wanted to explore was the patients’ presumed reactions of surprise and disappointment when referred directly to a psychologist instead of a doctor. One finding not expected by the authors was that no such results were found. The informants instead express their own intention to see a psychologist already when contacting the PHCC. Consequently, the innovative symptom-based sorting system introduced at the PHCC seems to provide that which patients already expect or at least do not question. Patients seem to be ahead of care institutions in their wish to be able to choose preferred occupational group themselves. There are no expressed hesitations about seeing a psychologist; fears are instead about *not* being given an appointment, characteristics of the psychologist, and what the outcome of the assessment will be. That which patients consider a positive surprise is how quickly and easily that appointment is accessed; here the triage offers something new that contradicts previous experiences or expectations. The results could be seen as in line with one of the present goals of the health-care system, to increase the patient's choice (NHS, 2010; Socialstyrelsen, 2010). That patients are active agents with their own intent is supported by other studies (Coulter, 2005). Patients emphasize the crucial importance that availability of quick psychological assessment has for the sensitive help-seeking process. Seeking help for mental health issues is considered a sensitive matter and people are reluctant to do so (Mojtabai *et al.*, 2002; Mojtabai, 2007; Schomerus *et al.*, 2012). Here the importance of short waiting time is important, also supported by other studies (Åsbring and Hochwälder, 2009). The triage can help lower the threshold to seek help for mental health issues, and thereby contribute to earlier detection.

As mentioned in the result section, the nurse is guided by the patients’ own wishes rather than symptoms. This supports the patients’ choice, which enhances motivation and compliance (Dwight-Johnson *et al.*, 2001). The patients’ experience of making the decision somehow contradicts the original intent of the triage model, where the nurse is the one deciding which occupational group to book. It could also be that the nurses so skilfully guide the patients, making them feel like it is their own incentive to see a psychologist. Or it could be that patients who ask to see a doctor are determined and hard to redirect to a psychologist, and therefore they are not present in the data. Finally, it could be that the nurses’ sorting task in the triage would go against the traditional nurturing role as described in the literature (Eley *et al.*, 2012).

There are rich accounts in the data about the experience of being assessed by a psychologist that may not be specific to the triage situation. Even before contact, patients have increased tension with both positive and negative expectations (DeFife and Hilsenroth, 2011). The patients wonder whether their symptoms are severe enough, and fear not being taken seriously. This is supported by a previous study on Swedish adolescents (Åsbring and Hochwälder, 2009) and is generally found when patients seek health care (Larsson *et al.*, 2009; Toye and Barker, 2011). Something that is emphasized when seeking help for mental issues is that it is experienced as sensitive and personal. This could explain the highly opinion-laden, often black or white, expressions in the data. Previous studies and psychological theory describe an increased tendency to both idealize and devalue when experiencing heightened mental stress (Kernberg, 1975) and that stress influences cognition (Währborg, 2002; Mather and Lighthall, 2012). This momentum can fuel the assessment outcome in a positive way, but also increases the risk for feelings of rejection or disappointment. The possibility of friction when meeting the health-care system is evident. Patients are aware of the possibility to see a psychologist, but may have less information about the limitations in the available treatment. This and other studies show a need for more specific information about the available treatment options in primary mental health care (Åsbring and Hochwälder, 2009; Kovandzic *et al.*, 2011). The disappointment in not being offered treatment that mounts up to one's experienced need may exist in all care-seeking situations (Larsson *et al.*, 2009). That said, it is the authors’ impression that there is an extra sensitiveness when seeking help for mental health issues, as these problems feel so closely connected to one's core self.

Recurring in the data is the patients’ experience of their needs not being sufficiently met. There is often a gap between patients’ demands and the financial and organizational limitations of health care. The triage expands the patients’ choice in relation to access and which occupational group to meet, but within regular restrictions concerning the available treatment. Perhaps this could explain some of the disappointment expressed by the patients. When individual choice and autonomy for patients increase (Edwards and Elwyn, 2009), the dilemma between free choice and limited resources is heightened, also discussed regarding increased patients’ choice in the UK National Health Service (Oliver and Evans, 2005; Samele *et al.*, 2007; Barr *et al.*, 2008).

### Implications for clinical practice

• The triage system seems to satisfy the patient's wish and need for quick access to a psychologist. By lowering the threshold to mental health care, the triage can contribute to detect mental health issues sooner. For the patient, to be able to choose the type of treatment also increases compliance and treatment effect.
• The finding that the triage is guided more by patients’ intentions than their symptoms could imply that some patients who might benefit from a psychological assessment miss out. To alter this, there may be a need for more guidance and decision making by the nurses. If so, more education or support to the nurse could be beneficiary.
• Patients seem to have wide and not always realistic expectations about the meeting with the psychologist, indicating a need for more information about the assessment and possible outcomes. This would increase the possibilities of the patient making an informed choice and knowing what to expect. To meet the patients’ expectations, there could also be a need for the psychologists to require further education and/or skill.

### Further research

• To develop the triage further, the nurses’ experiences and needs should be considered an important topic for research.
• The disappointment or dissatisfaction that patients express need to be studied further. Are there better ways to meet the patients’ demands? Or is some disappointment to be expected in any health-care situation?
• Research on psychological treatment is plentiful (Lambert *et al.*, 2004; Roth and Fonagy, 2005). Research on psychological assessment, in general, and on direct access to PHCC psychologists, in particular, is scarce. Being an important part of the psychologists’ work, this is an area of interest for further research.

### Strengths and limitations

The sample was representative of patients seeking health care for mental health issues in terms of gender (Clarkin *et al.*, 2004; Lambert *et al.*, 2004; Roth and Fonagy, 2005) and diagnosis (Socialstyrelsen, 2007). It can be argued that the results of qualitative data analysis is not generalizable to all situations; however, considering the adequate size (Kvale, 1996) and representativeness of the sample in this study, we find it possible to suggest that the findings could be applicable to similar groups in similar settings (Graneheim and Lundman, 2004).

The focus of the interviews was to cover the period of the triage. However, as some time had passed, it is possible that the way the patients experienced the process following the assessment might influence how the triage is looked back upon. In trying to minimize the impact of patients’ general attitudes to the PHCC and possible future care, the interviews were conducted by a person not connected to the PHCC, at a location outside the clinic.

The first authors were themselves psychologists at the setting, giving them a first-hand understanding of the informants’ descriptions. Working close to the setting for the study can be seen as a prerequisite and a necessity when doing clinical research in a naturalistic environment. The authors strived to distance themselves from a clinical reading of the data and instead interpret the text within a qualitative research process (Graneheim and Lundman, 2004). Being more than one author with complementary and diverse perspectives contributed to illuminating the data (Table 2).

**Table 2:** Example of data analysis

Triage	Meaning unit	Code	Category	Theme
Contacting	‘No, but you could say it's sort of a little conflicting feelings from the moment you enter, so to speak… on the one hand you want to go there and on the other hand you feel like it's hard to do it’	Building up strength to seek help	To seek help for mental health issues is demanding	Appreciating quick access as seeking help is demanding
Booking	‘I wanted to talk to a psychologist, that is why I called, though I didn′t really have many expectations, I wasn't really sure how it worked, I had only heard that there were psychologists here at the health center’	Knew there was a psychologist, wanted it for himself or herself	Patients are informed active agents	The nurse responds to the patient's own intention
Assessment	‘well…that was really the… I was thinking and then the actual conversation, it was still…it was really just the time that was a nuisance… that I didn't have time to start talking about other things …well, there wasn't enough time really…’	Wanted the assessment to be longer	Disappointing discrepancy between experienced need and offered care	Relief and disappointment when diverse expectations meet the structured assessment
